# Vascular inflammation on a chip: A scalable platform for trans-endothelial electrical resistance and immune cell migration

**DOI:** 10.3389/fimmu.2023.1118624

**Published:** 2023-01-24

**Authors:** Haley Ehlers, Arnaud Nicolas, Frederik Schavemaker, Jeroen P. M. Heijmans, Martin Bulst, Sebastiaan J. Trietsch, Lenie J. van den Broek

**Affiliations:** ^1^Mimetas B.V., Leiden, Netherlands; ^2^Leiden Academic Centre for Drug Research, Leiden University, Leiden, Netherlands; ^3^Sciospec GmbH, Bennewitz, Germany

**Keywords:** vascular inflammation, immune cell migration, TEER, organ-on-a-chip, endothelium, TNFα, INF-γ and OrganoPlate

## Abstract

The vasculature system plays a critical role in inflammation processes in the body. Vascular inflammatory mechanisms are characterized by disruption of blood vessel wall permeability together with increased immune cell recruitment and migration. There is a critical need to develop models that fully recapitulate changes in vascular barrier permeability in response to inflammatory conditions. We developed a scalable platform for parallel measurements of trans epithelial electrical resistance (TEER) in 64 perfused microfluidic HUVEC tubules under inflammatory conditions. Over 250 tubules where exposed to Tumor necrosis factor alpha (TNFα) and interferon gamma (INF-γ) or human peripheral blood mononuclear cells. The inflammatory response was quantified based on changes TEER and expression of ICAM and VE-cadherin. We observed changes in barrier function in the presence of both inflammatory cytokines and human peripheral blood mononuclear cells, characterized by decreased TEER values, increase in ICAM expression as well changes in endothelial morphology. OrganoPlate 3-lane64 based HUVEC tubules provide a valuable tool for inflammatory studies in an automation compatible manner. Continuous TEER measurements enable long term, sensitive assays for barrier studies. We propose the use of our platform as a powerful tool for modelling endothelial inflammation in combination with immune cell interaction that can be used to screen targets and drugs to treat chronic vascular inflammation.

## 1 Introduction

Acute inflammation is an essential mechanism to ward off infection and support healing of injuries. Autoimmune disorder or repeated injuries can impair the immune system’s ability to regulate the inflammatory response, increasing the risks of inflammation becoming chronic and detrimental (1). Vasculature plays a crucial role in inflammatory processes by transporting immune cells, cytokines and chemokines throughout the body (2). Chronic inflammation can affect the vasculature and is associated with increased risk of cardiovascular diseases like endothelial dysfunction and atherosclerosis (3). Unfortunately, cardiovascular diseases are one of the leading causes of deaths worldwide, taking approximately 17.9 million lives a year (4).

The endothelium is responsible for many functions like vascular tone, barrier function, injury repair, and metabolism (5). Endothelial dysfunction is characterized by an increase in permeability of the vessels and endothelial activation (6). In acute inflammation, barrier leakage is brief, but in chronic inflammation this leakage is sustained and the vasculature remodels to an activated leaky phenotype (7). In order to maintain healthy barriers, endothelial barrier comprise adherent junctions, tight junctions and gap junctions. VE-Cadherin is an adherent junction that is the main component in endothelial junctions and is essential for regulation of endothelial barrier (8). Pro-Inflammatory cytokines like tumor necrosis factor alpha (TNFα) and Interferon gamma (INF-γ) are known to cause endothelial cell activation (9, 10) and can lead to VE-Cadherin destabilization (11, 12). This endothelial activation leads to increased expression of various adhesion proteins like ICAM (5, 6, 13). Phosphorylation of VE-Cadherin (14, 15) allows for the migration of immune cells out of the vessel into the surrounding arterial tissue. While VE-Cadherin plays a critical role in vascular permeability and immune cell migration (16), the exact relationship between immune cell migration and vascular leakage is not fully understood (13, 17). Therefore, there is a need of *in vitro* models that properly recapitulate the vascular barrier and methods that quantify the barrier integrity of these models.

The development of *in vitro* models of vascular barrier function has been facilitated by the development of 3D culture and microfluidic organ-on-a-chip technologies (18, 19). Vasculature-on-a-chip models have several benefits over conventional *in vivo* and cell culture methods, allowing for research on vessel formation and creation of *in vitro* models for changes in barrier (20–22). When modeling vasculature *in vitro*, important parameters to take into account include mechanical, chemical and biological factors that can be incorporated in the microfluidic models (23). Organ-on-a-chip models have previously been developed to study specific vascular diseases including atherosclerosis, radiation injury, and thrombosis (24).

**Figure 1:**
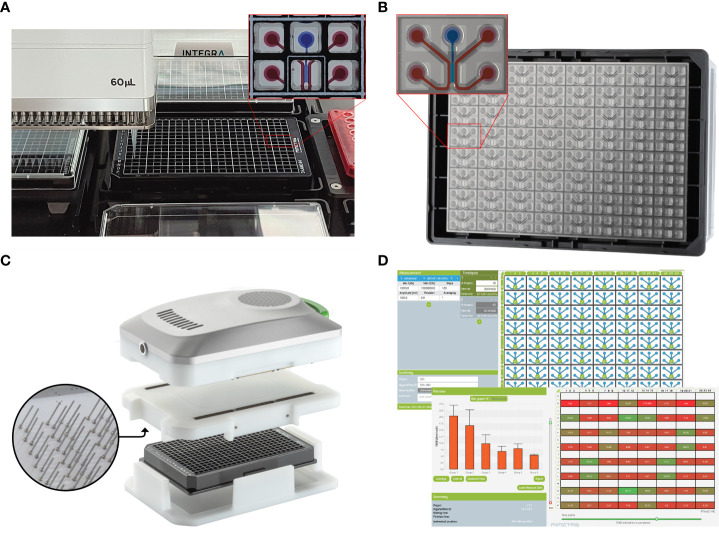
A screening platform for automated seeding of an organ-on-a-chip endothelial model combined with high throughput TEER measurements. **(A)** A OrganoPlate 3-lane 64 tissues in a standardized 384-titerplate format. Here, the Organoplate 3-lane 64 seeded on a Biomek i5 automated liquid handling platform. The expanded portion of the plate highlights the top view of one individual chip covering six microtiter wells. One microfluidic chip is enlarged to show the 3 individual microchannels. The perfusion channels used for vessel formation are filled with red dye and the the gel channel is filled with blue dye. **(B)** The bottom view of the OrganoPlate 3-lane 64 highlighting the microfluidic channels, making up 64 individual chips attached to the bottom of the microtiter plate, allowing for high content imaging of each individual chip. One microfluidic chip is enlarged to highlight the 3 individual microchannels. The perfusion channels used for vessel formation are highlighted in red and the gel channel is highlighted in blue. **(C)** The OrganoTEER, a commercially available automated TEER measurement system compatible with previous OrganoPlate 3-lane 40 based tubular models (28). **(D)** OrganoTEER software used to perform TEER measurements and perform automated analysis of the results on an OrganoPlate 3-lane 64.

We previously reported the capability of organ-on-a-chip assays for the formation of vascular models (25), including T cell migration (26) and monocyte adhesion (27) in the OrganoPlate. This platform integrates dozens independently addressable microfluidic circuits integrated into a laboratory standard 384 well microtiter plate (**Figures 1A, B**). By guiding extracellular matrix (ECM) gels into a microfluidic channel using capillary barriers called phaseguides (29), we could create independently accessible microfluidic channels on either side of an ECM scaffold. Once the endothelial cells have grown to form a tubule in the microfluidic channel and against the ECM gel, assays such as barrier analysis or the addition of PBMCs to the endothelial tubule can be performed (30–32).

Changes in vascular barriers were measured using a fluorescent dextran assay (25). However, trans endothelial/epithelial electrical resistance (TEER) is used as the gold standard for assessing barrier function *in vitro* and has the potential to avoid the use of an added fluorescent probe and offer increase time resolution. Electrical impedance is correlated with biological aspects such as cell layer confluency, morphology and paracellular junction composition. TEER measurements are typically used in the context of 2D cell cultures, either in an insert-based culture device (33, 34) or directly onto a patterned electrode substrate (35). Such measurements have been used to interrogate epithelial and endothelial tissues (36, 37). We previously reported a method and instrument for rapidly and automatically measuring the TEER values of 40 independent chips in the OrganoPlate 3-lane 40 to study disease processes in gut on a chip models comprising immortalized cell lines or IPSC derived cells (28, 38, 39). However, *in vitro* models of endothelial barriers typically show much lower TEER values than other epithelial models (33) and thus require a significant increase in the sensitivity of instruments used for TEER measurements.

In this paper, we developed a scalable and real time method to study vascular barrier function by combining the speed and sensitivity of TEER based measurements with an automation friendly organ-on-a-chip platform. Due to its standard 384 well titer plate format, optically clear glass and a fixed microfluidic height of 220 µm, the OrganoPlate is readily compatible with high content microscopy, confocal microscopy and is suitable for performing multiple high throughput assays on a single chip (40). We previously reported methods for the formation of endothelial tubules in the for angiogenic sprouting (41) and the use of the platform to study T cell migration (26) in Organoplate based 3D microfluidic channels. Here we used the OrganoPlate 3-lane 64 (**Figure 1B**), which allows for extracellular matrix (ECM) seeding in the middle channel and growth of endothelial tubules against the gel. Thanks to its two by three wells pitch, it is well suited for automation workflows (**Figure 1A**). We developed a 3-lane 64 OrganoPlate based TEER measurements apparatus that can be used inside an incubator for long term permeability studies of 64 separate tubules (**Figures 1C, D**). This combination of TEER and the OrganoPlate 3-lane 64 offers real time interrogation of up to 64 perfused endothelial models exposed to targeted apical and basal cues in a membrane free, ECM supported microenvironment. This paper shows the use of scalable, automatable OrganoPlate based model of vascular inflammation comprised of up to 64 HUVEC endothelial tubules per plate including cytokine treatment and/or to human Peripheral Blood Mononuclear Cells (PBMC) in combination with continuous TEER measurements. We demonstrate the capability for multiplexing readouts, such as barrier, phase contrast imaging and immunofluorescence, for recapitulating vascular inflammatory processes that can be used in drug development or drug delivery studies.

## 2 Methods

### 2.1 Cell culture

HUVECs (Lonza, C2519AS) were thawed and cultured in EGM-2 medium (Lonza, CC-3162). HUVECs were frozen at passage number 5 in EGM-2 + 10% FBS + 10% DMSO and stored at -150oC for further use. Peripheral blood mononuclear cells (PBMCs) were isolated from a Buffy Coat (Sanquin) as previously described by de Haan et al. (26) PBMCs were banked in 90% FBS + 10% DMSO and stored at –150°C for further use.

### 2.2 PBMC stimulation and labeling

PBMCs were thawed and cultured in AIM-V medium (Gibco, 12055-091). T cell population in the PBMCs were stimulated for 48 hours by Dynabeads^®^ Human T-Activator CD3/CD28 (ThermoFisher Scientific, 11131D) according to the manufacturer’s instructions. Unstimulated PBMCs were cultured in AIM-V for 48 hours. Prior to adding PBMCs to endothelial tubules in an OrganoPlate, PBMCs (both unstimulated and stimulated) were labeled with CellTracker Orange CMRA (Invitrogen, C34551), as previously described in de Haan et al. (26) In short, CMRA stock of 5 mM in dimethylsulfoxide (DMSO, Sigma, D8418) was diluted to a working concentration of 2.5 µM in AIM-V medium. PBMCs were harvested and pelleted (300g for 5 min) before being resuspended in 2 ml of CMRA working solution. Cells were then placed in a humidified incubator at 37°C, 5% CO_2_ for 30 minutes. After the incubation period, 10 mL of AIM-V medium was added, cells were counted, and cells were pelleted (300g for 5 min). The appropriate volume was added to the PBMCs to have a final concentration of 400,000 cells/ml.

### 2.3 OrganoPlate culture

OrganoReady Blood Vessel HUVEC 3-lane 64 plates (Mimetas B.V, MI-OR-BV-02) were cultured according to manufacturer’s instructions. Medium was changed on day of receiving to OrganoMedium HUVEC-BM (Mimetas B.V.). OrganoReady Blood Vessel HUVEC 3-lane 64 are ready to use HUVEC tubes in OrganoPlates, that follow a similar process for ECM seeding (rat tail collagen 1 at 4mg/ml), endothelial cell seeding and establishment of perfusion through the tubules as described by Duinen et al. for the OrganoPlate 3-lane 40 (41). Perfusion flow was maintained by placing the plate on OrganoFlow rocker (Mimetas B.V., MI-OFPR-L) set at 14 degrees with 8-minute intervals optimized for the 3-lane 64. This allows the perfusion of medium through the endothelial tube. On the second day after receiving, cultures were exposed to cytokines or PBMCs. Prior to TEER timelapse set up, the OrganoPlate was transferred to the ImageXpress Micro XLS microscope (Molecular devices) and every individual chip on the OrganoPlate 3-lane 64 was imaged for 4x magnification phase contrast images. OrganoPlate was placed back to 37°C incubator. TEER timelapse were started directly after addition of medium.

### 2.4 Electrode board interface for the 3-lane 64 OrganoPlate

New electrode boards matching the layout of the 3-lane 64 (**Figure 1**), comprising 512 stainless steel electrodes were designed and manufactured as previously reported (28). Prior to measurements, the electrode board was connected to the OrganoTEER measurement unit using mezzanine connectors and inserted into the inlet and access wells of the OrganoPlate to provide one current-carrying loop and one voltage-sensing loop for each of the 64 chips through a total of 512 electrodes.

### 2.5 Cytokine/PBMC exposure and TEER timelapse

Prior to all experiments, the OrganoTEER electrode boards were disinfected by spraying the electrodes surface with 70% ethanol and allowed to dry in a sterile flow cabinet. Before the start of timelapse measurements, a single electrode board and measurement unit were assembled and inserted into a sterile single well plate. The stack was then placed in an incubator at 37°C for at least two hours to allow the OrganoTEER temperature to equilibrate.

The left channel inlet and outlet of each chip were filled with 50 μL of HBSS (for the cytokine exposure study) or basal medium (for the PBMC study) 1 hour prior to starting timelapse. For the PBMC study, the left 4 columns of chips had basal medium added to the left perfusion lane and the right 4 columns of chips had basal medium with 800 ng/ml of CLCX12 to the left perfusion lane. The incubated OrganoTEER was placed into the flow cabinet and the single well plate was replaced with the OrganoPlate. The OrganoTEER was placed back into the incubator and onto the OrganoFlow rocker for 30 minutes prior to the timelapse. The OrganoFlow rocker was set to a static horizontal position for 6 minutes to let the liquid levels within the wells equilibrate, after which the T_-1_ TEER measurement was taken.

For the cytokine exposure, TNFα (R&D Systems, 210-TA-020) and INF-γ (R&D systems, 285-IF-100) were diluted in OrganoMedium HUVEC-BM to the appropriate concentrations (0.1, 1, 10 and 100 ng/ml). The medium was equilibrated to 37°C for at least 30 minutes before addition of 50 µl medium to inlets and outlets of each chip in the OrganoPlate.

For the experiments with PBMCs, PBMCs were harvested and prepared as mentioned above, for the addition of 20,000 cells/chip. For conditions with cytokines (10 ng/ml of TNFα and INF-γ), 2x concentration (20 ng/ml) of cytokines were added to medium. To ensure swift media change, the different medium conditions were pipetted in a 384 well plate according to the inlet and outlet layout of the OrganoPlate, and allowed to incubate for 15 minutes at 37°C. The conditions where arranged such that 50 µl of medium containing 20,000 PBMCs were added to the corresponding OrganoPlate inlets and 50 µl medium with or without cytokines were added to the corresponding OrganoPlate outlets.

To expose the tubules to their respective condition, the medium was aspirated from all inlet and outlet wells of the OrganoPlate. A multi-channel pipette was used to transfer 50 μL of medium containing cytokines or PBMCs from the 384 well plate to the corresponding wells of the OrganoPlate. To minimize temperature fluctuation, the OrganoTEER assembly was placed on the static rocker platform. The first TEER measurement was taken immediately after the medium change. The OrganoFlow was started 30 seconds after the completion of the first timepoint measurement (14 degrees changes every 8 minutes). To ensure synchronicity with the rocker cycle, TEER measurements were taken every subsequent 4 minutes for the first 44 minutes (16 points), then every 64 minutes for 43 hours. An endpoint measurement was taken between 44 to 48 hours. After the completion of the TEER timelapse, the OrganoPlate was removed from the TEER device and transferred to the microscope and phase contrast images of each chip were taken. After phase contrast imaging, the plates were fixed in 3.7% formaldehyde (Sigma, 252549) and stored at 4°C.

### 2.6 Data acquisition and extraction

All measurements were composed of 121 frequency dependent impedance points equally distributed in a logarithmic scale from 1000 Hz to 1 MHz. Using a controlled voltage source linked to an operational amplifier, the OrganoTEER imposed a sinusoidal AC voltage of 100 mV across the current carrying electrodes. The resulting current was measured across voltage sensing electrodes *via* a transimpedance amplifier. Within the measurement unit, a set of 12 separate digital impedance analyzer units linked to multiplexing units was used to acquire impedance values of 12 chips at a time.

### 2.7 Data compensation and fitting

To minimize the impact of parasitic capacitance (See **Supplementary Information 1**), we built three compensation boards by replacing the electrodes of each chip connection by a resistor of defined value per board (24 kΩ, 30 kΩ and 36 kΩ respectively, E24 standard). Each compensation board was measured once in the incubator where measurements were performed. The compensation spectra and associated resistor values were used by the OrganoTEER software to compensate the loss of high frequency signal induced by parasitic capacitances. All compensated measurement spectra were then automatically fitted against an analog model using the OrganoTEER fitting algorithm (28). The extracted barrier resistance parameter, measured in Ω, was multiplied by an approximative value of the ECM-cell interface area (0.0057 cm^2^) to produce a TEER value per chip in Ω·cm^2^.

### 2.8 Immunocytochemistry

After fixation, cultures in the OrganoPlate were stained for immunofluorescent markers. As described previously, in short, cells were permeabilized using a Triton X-100 solution for 10 min and blocked using a buffer containing FBS, bovine serum albumin, and Tween-20 for 45 min (42). Primary antibody was incubated for 1–2 hours or overnight, after which secondary antibody was incubated for 1 hour. The following primary antibodies were used to stain fixed cultures: Anti‐VE-Cadherin 1:500 (Abcam, ab33168), anti‐ICAM‐1 1:50 (R&D systems, BBA3), anti-human CD45 (R&D systems, MAB1430). The following secondary antibodies were used to stain fixed cultures: Goat anti‐rabbit IgG (H+L) Alexa Fluor 488 1:250 (Thermo Fischer Scientific, A11008), Goat anti‐mouse IgG (H+L) Alexa Fluor 647 1:250 (Thermo Fischer Scientific, A21428) and CF647 Goat anti‐mouse IgG (H+L) Alexa Fluor 647 1:250 (Biotium, 20040). Nuclei were stained using Hoechst (ThermoFisher, H3570). After staining, the OrganoPlate was transferred to a confocal high content imaging microscope for automated imaging (Micro XLS-C, Molecular Devices). Images were acquired at 10x magnification at 3 µm increments along the height of the microfluidic channel. Analysis was based on Sum Projection (ICAM expression) or Max projection (VE cadherin) images of the top and bottom 10 z-slices.

### 2.9 Image analysis

Quantification of PBMC dynamics and ICAM immunofluorescent staining was performed as previously reported by de Haan et al. in 2021 (26). Quantification of VE-Cadherin immunofluorescent staining was performed using machine learning based segmentation software Cellpose2 (43) and image processing software Fiji (44). The cell perimeter was segmented based on VE-Cadherin expression, using the built in livecell LC4 model. The segmented cell outlines were loaded into Fiji and roundness value for each outline was extracted using the built in Particle analysis tool.

### 2.10 Statistical analysis

Means of two or more groups were assessed using Brown-Forsythe and Welch ANOVA (Gaussian, heterogeneity of variance) or Kruskal–Wallis tests (non-Gaussian) for statistically significant differences. In the case of two or more factors, two-way ANOVA tests or three-way ANOVA tests were formed depending on the number of factors. Multiple comparisons were accounted for using Tukey’s or Dunnett’s tests. All data set were tested for normality using QQ-plots and residuals for normality. Statistical analyses were performed using GraphPad Prism v9.4 (GraphPad Software). Differences were considered significant when *p* < 0.05. For the number of replicates, each independent experiment is represented by a “N” and the individual chip is represented by a “n”. Total number of replicates is N x n.

## 3 Results

### 3.1 Enabling TEER measurements in the OrganoPlate 3-lane 64

**Figure 2:**
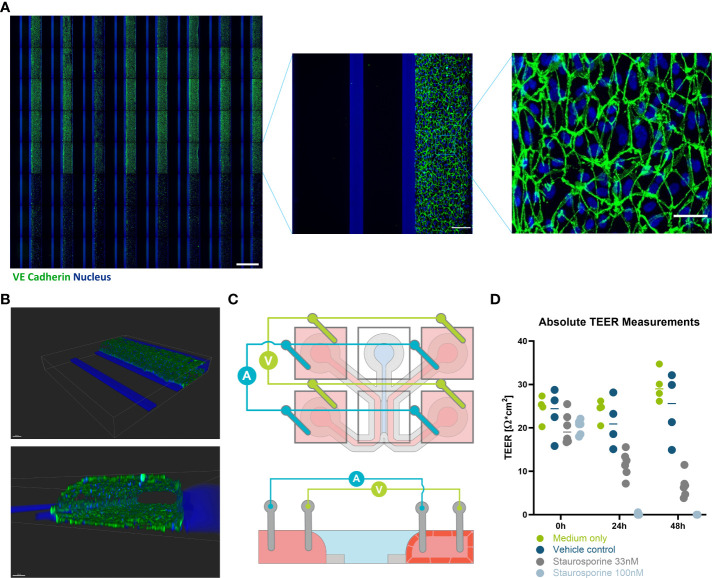
Immunofluorescent staining and TEER measurements of HUVEC endothelial tubules in OrganoPlate 3-lane 64. **(A)** Montage of Immunofluorescent images (VE-cadherin (green) and nucleus (blue)) of 64 HUVEC endothelial tubes (right perfusion channel) cultured against a collagen I ECM layer (center channel) in the OrganoPlate 3-lane 64 (scale bar 1000 µm). Bottom three rows were Staurosporine treated. One chip from the montage is blown up to illustrate how the chip looks (scale bar 150 µm). Another zoom of the endothelial tube is shown to demonstrate the cell morphology (scale bar 50 µm). **(B)** Confocal reconstruction of a HUVEC tubule using VE-Cadherin (green) and DAPI (blue) staining. **(C)** Schematic illustration of how a microfluidic chip and the positioning of the TEER electrodes. Both current carrying and voltage sensing loops are formed across the gel and perfusion channel *via* four pairs of electrodes, shorted pairwise in the inlets and outlets of their perfusion channels. The bottom schematic depicts a side view of the chip in the X-Z plane showing how the endothelial cells will grow to form a tubule against the gel, while the left channel remains empty. **(D)** values of the HUVEC tube at 0, 24, and 48 hours with the addition of Staurosporine to interrupt the barrier (n= 4-6). Scale bars are 100µm.

The OrganoTEER platform was optimized for compatibility with the automation friendly OrganoPlate 3-lane 64 and sensitivity was improved to increase its dynamic range around typical TEER values of endothelial culture. We redesigned the OrganoTEER system for the measurement of single tubules in the OrganoPlate 3-lane 64. We built an Electrode board compatible with both the OrganoPlate 3-lane 64 and the OrganoTEER measurement unit (**Figure 1B**). The OrganoTEER software was rewritten to accommodate the setup and measurement of each 64 chips (**Figures 1C, D**). The system allowed 512 stainless steel electrodes to be inserted into each chip perfusion channels access wells. For each chip, one current-carrying one voltage-sensing loop was created from the inlet and outlet of one perfusion channel to the outlet of the opposite perfusion channel (**Figure 2C**). For both the current carrying and voltage sensing loop, each point of the 4-terminal setup was shorted to a pair of electrodes inserted in both inlet and outlet of the same perfusion channel. A typical measurement takes under 2 minutes for an entire plate. The Software allows timelapse measurements to be configured to accommodate long term exposure over several days.

The configuration of our TEER measurement setup increased the chip electrical resistance over the 3-lane 40 due to longer current path. The electrode pairs on the basolateral side were inserted in the perfusion inlet and outlet in the 3-lane 64. This induced a higher impact from parallel parasitic elements. To compensate for this, we built three separate electrical compensation boards using the PCB layout the 3-lane 64 electrode board. In place of the electrodes, we soldered surface mounted resistors electrically connecting the reference and counter side to the work and working sense side. The boards resistances were 24 kΩ, 30 kΩ and 36 kΩ. Prior to the exposure experiment, we measured the impedance spectra of each of the 3 compensation boards, which we then used to perform a multiple load compensation on all measured data prior to fitting using the OrganoTEER software (**Supplementary Information Figures 1, 2**).

### 3.2 Evaluation of HUVEC tubule in 3-lane 64

OrganoReady Blood Vessel HUVEC 3-lane 64 contained 60 tubular structures were purchased. The endothelial cells in these tubules had a cobblestone like appearance that can be observed using phase contrast microscopy throughout the duration of the culture (**Supplemental Figure 3A**). These endothelial cells expressed VE-cadherin at the junctions between cells (**Figure 2A**). Using confocal microscopy, a 3D representative image was created showing that the endothelial cells formed a confluent 3-dimensional endothelial tubule (**Figure 2B**). Using the new TEER board set up described above, the lower resistance endothelial tubule was measured and disruptions in barrier due to Staurosporine could be detected (**Figure 2D**).

### 3.3 Cytokine induced vascular inflammation

**Figure 3:**
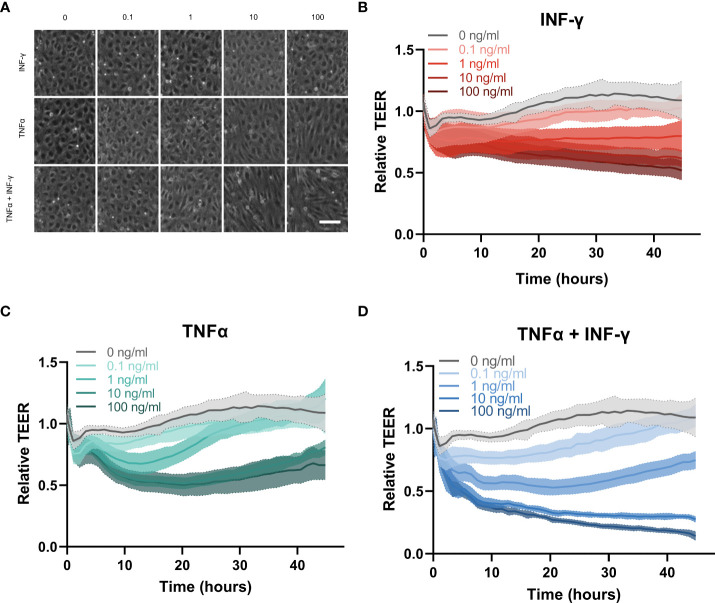
Cytokine response of HUVEC endothelial tubes in the OrganoPlate 3-lane 64. **(A)** Representative phase contrast images of zoom in of endothelial vessel exposed to inflammatory triggers for 44hrs. Concentration of TNFα and INF-γ in ng/ml. Scale bar is 100µm. **(B–D)** Relative TEER timelapse of 44 hours of exposure to increasing concentrations of **(B)** INF-γ, **(C)** TNFα and **(D)** combination of TNFα and INFγ (N=3, n=3-5 per experiment).

To model vascular inflammation, the endothelial vessels were exposed to different inflammatory cytokines for 48 hours. Phase contrast images showed that the vessels exposed to INF-γ had minimal changes in morphology. However, as the concentration of TNFα increased, the endothelial cells showed a less rounded and aligned phenotype (**Figure 3A**). These changes in morphology were more pronounced in the combination of cytokines compared to TNFα alone.

The effect of inflammatory cytokines on TEER was monitor over 44 hours. INF-γ showed a dose dependent slow decrease in barrier integrity over time (**Figure 3B**). TNFα induced a sharp decline in barrier integrity around 2 hours which recovered towards the end of the exposure (**Figure 3C**). The higher concentrations of TNFα at 10 and 100 ng/ml induced the same amplitude of barrier leakage with no significant difference, indicating that the increasing concentration of TNFα did not cause increased disruption beyond 10 ng/ml. The lower concentrations of TNFα (0.1 and 1 ng/ml) showed more dose dependent disruption. However endothelial barriers were fully recovered by the end of the exposure at which point their TEER was not statistically significantly different compared to medium control (**Supplementary Figure 2B**). The combination of INF-γ and TNFα had the largest effect on the barrier integrity of the endothelial vessel (**Figure 3D**). The combination of cytokines induced a dose dependent decrease in barrier integrity around 2 hours, similar to the changes in barrier due to TNFα only. However, the barrier did not recover for the remainder of the exposure as seen for the TNFα alone, except for the lowest concentration (0.1 ng/ml) which was not significantly different than the medium control at the final timepoint (**Supplemental Figure 4C**).

**Figure 4:**
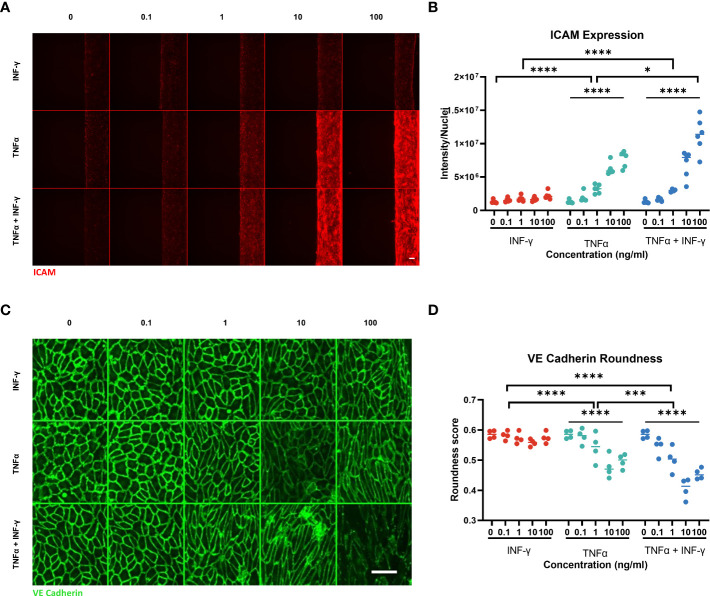
ICAM-1 and VE-Cadherin staining of endothelial vessel. **(A)** ICAM-1 expression staining of endothelial vessel exposed to TNFα or/and INF-γ in ng/ml for 44hrs. Scale bar is 100µm. **(B)** Quantification of ICAM-1 staining. Significant difference between cytokine conditions (*p<0.05, ****p < 0.0001) as well as a dose dependent effect for TNFα and TNFα + INF-γ (****p < 0.0001) Data was analyzed using Two-way ANOVA tests, followed by a Dunnet’s multiple comparison tests and Tukey’s multiple comparison test. **(C)** Montage of the max projection image of VE-Cadherin staining of the bottom 10 z-steps (3µm step size). Scale bar 100µm. **(D)** Quantification of cell roundness determined from VE-Cadherin staining. Significant difference between cytokine conditions (***p=0.0002, ****p <0.0001) as well as a dose dependent effect for TNFα and TNFα + INF-γ (****p < 0.0001). Data was analyzed using Two-way ANOVA tests, Dunnet’s multiple comparison tests and Tukey’s multiple comparison tests. (N=2, n=3-6) Scale bars are 100µm.

The two inflammatory cytokines also had a different effect on ICAM-1 expression. TNFα and combination of TNFα and INF-γ caused a significant dose dependent increase in ICAM-1 expression (**Figures 4A, B**). INF-γ only did not cause a significant increase in ICAM-1 expression. In addition to changing the ICAM-1 expression, TNFα and INF-γ affected the VE-cadherin expression differently. In the control conditions, the HUVEC cells showed a rounder and more cobblestone like appearance (**Figure 4C**). INF-γ did not have a strong effect on cell morphology and VE-cadherin expression. As the concentration of TNFα increased, the endothelial cell morphology was less rounded and slightly more aligned (**Figures 4C, D**). At the higher concentrations with TNFα and both cytokines, the endothelial tubes are not only less round, but the consistency of the VE-cadherin staining is reduced (**Supplemental Figure 3B**). The combination of cytokines increased these changes in VE- cadherin expression with decreased roundness of cells and less consistent VE-cadherin expression in a dose dependent manner.

### 3.4 Effect of immune cells on vascular inflammation

**Figure 5:**
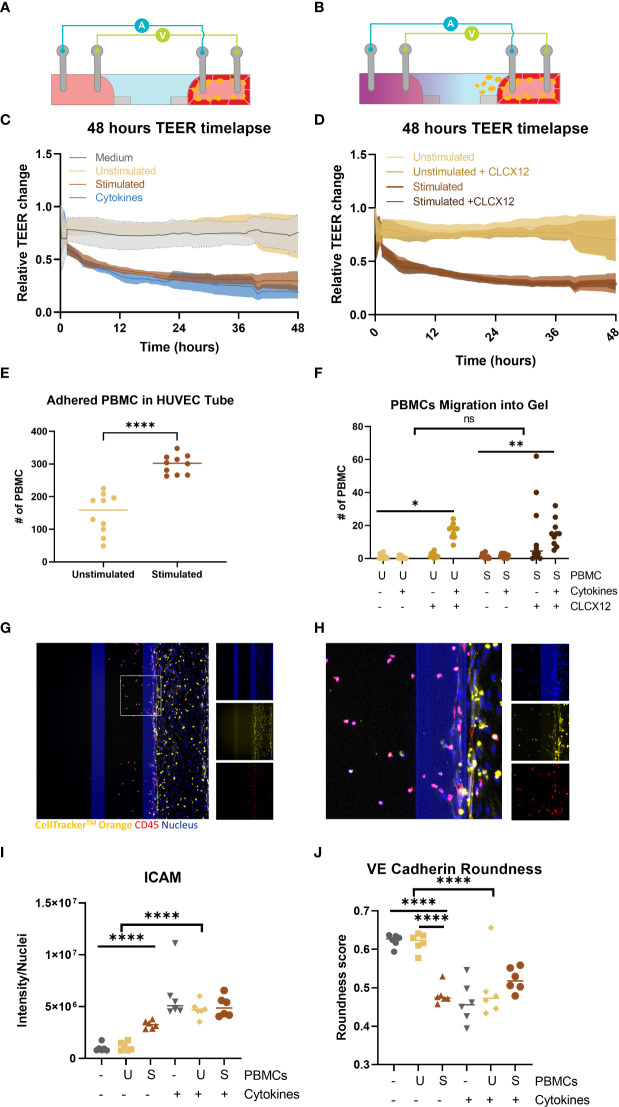
Perfusion and extravasation of peripheral blood mononuclear cells (PBMCs) through a HUVEC tubule into an ECM gel in an OrganoPlate 64. **(A)** Schematic of TEER electrodes with endothelial tubule and PBMCs adhered to the endothelial tube **(B)** Schematic of TEER electrodes with endothelial tubule and PBMCs migrating from the endothelial tube into gel channel due to addition of chemokine (purple) in basal lateral channel. **(C)** Relative change in barrier of PBMCs or 10 ng/ml of TNFα + INF-γ can be observed in the 48h timelapse. **(D)** TEER timelapse comparing conditions with and without CLCX12. No difference was observed between comparable conditions with +/- CLCX12. **(E)** Number of PBMCs adhered to the HUVEC tubules 48 hour after addition. Significant difference unstimulated and stimulated PBMCs (****p < 0.0001) was analyzed using Welch’s T tests. **(F)** PBMC migration out of endothelial vessel into gel channel. Significant difference between PBMC and PBMCs with cytokines and CLCX12 (* p=0.04, ** p < 0.0018) was analyzed using Brown-Forsythe and Welch ANOVA tests and Dunnett’s T3 multiple comparison test. 3way ANOVA showed no significant (ns) difference between unstimulated and stimulated PBMCs in migration of PBMCs into the ECM. **(G)** Fluorescent based image of immune cells perfused through a HUVEC tubule and migrated into ECM. **(H)** Area of interest from **(G)** to highlight PBMC migration and staining. Shown are nucleus (blue), CellTracker™ Orange stain (yellow) and CD45 (red). **(I)** ICAM-1 quantification after exposure and addition of PBMCs. Significant difference due to addition of cytokines (****p < 0.0001) as well an effect of addition of stimulated PBMCs without cytokines (****p < 0.0001) Data was analyzed using Two-way ANOVA tests with Šídák’s multiple comparisons test. **(J)** Quantification of cell roundness determined from VE-Cadherin staining. Significant difference due to addition of cytokines (****p < 0.0001) as well a significant different between medium and stimulated PBMC and unstimulated PBMCs and stimulated PBMCs (****p < 0.0001). Data was analyzed using Two-way ANOVA tests with Tukey’s multiple comparisons test. (N=2, n=3-6).

To determine whether we could also study the effect of immune cells directly on vascular inflammation non stimulated and stimulated PBMCs were added in the lumen of the endothelial tubules. The addition of immune cells to the endothelial tubule did not interfere with the addition of the TEER electrodes as observed in the schematic (**Figure 5A**). Addition of unstimulated PBMCs did not influence the endothelial barrier when compared to the control conditions (**Figure 5B**). However, stimulated PBMCs had similar effect on the barrier as the 10 ng/ml of TNFα and INF-γ condition. In both conditions, there was a sharp decrease in the first 2 hours that remained for the rest of the exposure. Stimulated PBMCs adhered to the vessel wall in greater number than unstimulated PBMCs (**Figure 5C**).

The chemoattractant, CLCX12, was added to the empty channel to create a chemotactic gradient to induce immune cell migration (**Figure 5D**). The addition of CXCL12 did not result in a significant change in barrier integrity of the vessels that were perfused with stimulated or unstimulated PBMCs (**Figure 5E**). Addition of only CXCL12 did not result in significant increase in adhesion (**Supplemental Figure 5B**) or extravasation of PBMCs (**Figure 5F**). The combination of CLCX12, TNFα and INF-γ resulted in a significant increase in migration of both unstimulated and stimulated PBMCs out of the vessel into the ECM (**Figure 5F**). PBMC migration into the gel channel was confirmed by the overlapping of CD45 and CellTracker™ Orange (**Figures 5G, H**).

Adding stimulated PBMCs to the endothelial tubule caused a significant increase in ICAM-1 expression in the endothelial tubule when compared to the medium only or unstimulated PBMCs (p<0.00001) (**Figure 5I** and **Supplemental Figure 5E**) However when cytokines were added all conditions showed even higher ICAM-1 expression compared to stimulated PBMCs (**Figure 5I**). A significant effect on cell roundness by VE-cadherin expression was found by the addition of stimulated PBMCs (**Figure 5J**). The addition of cytokines did not have an added effect on cell roundness when compared to stimulated PBMC condition (**Figure 5J** and **Supplemental Figure 5D**). When both stimulated PBMCs and cytokines were added the VE-cadherin expression was almost gone (**Supplemental Figure 5D**).

## 4 Conclusion and discussion

Endothelial dysfunction induced by chronic inflammation is a precursor to atherosclerosis and is characterized by increased barrier permeability and immune cell migration. We have demonstrated an organ-on-a-chip platform capable for modeling and quantifying endothelial response to inflammation. We adapted the commercially available OrganoTEER system to enable simultaneous TEER measurement of 64 perfused endothelial tubules. Using our compensated TEER measurement assay, we showed significant dose dependent effect on endothelial barrier of different inflammatory cytokines triggers over a 48-hour time period. INF-γ, TNFα and their combination showed a significant decrease in endothelial barrier within four hours for all tested concentrations. We showed the effect of perfused immune cells on the endothelial barrier. In addition to following the endothelial barrier function over time, we multiplexed our assay with immunostaining-based assays to show immune typical expression of ICAM-1 and changes in cell morphology using VE-Cadherin. These findings demonstrate the capability to study how different compound or cytokines affect endothelial barriers and how they influence the immune endothelium interaction.

We adapted the OrganoTEER electrode board and data compensation method for the precise measurement of 64 endothelial tubules. With a total of 9940 TEER measurements in our monoculture cytokine exposure (160 chips over 44 hours) and 5580 measurements in our immune migration experiment (98 measured chips over 44 hours), this paper represents, to our present knowledge, the largest published TEER dataset on an organ-on-a-chip model. The ability to continuously assess the permeability of each individual tubule provided insight in the dynamics of the tested inflammation triggers. With such data density, we were able to observe concentration dependent transient effects, such as recovery of our TNFα exposed monocultures, which might not have been seen with larger measurement time intervals. The scalability of the system allowed us to use high numbers of replicates (between 7 and 14 depending on conditions) increasing the confidence in our results. This throughput could also be leveraged for large scale screening studies on an organ-on-a-chip platform. The small footprint of OrganoTEER system make it fast and easy to use in a manual, medium throughput setting, but could also be beneficial for integration in a robotic environment.

Due to the high background electrical resistance associated with the microscopic channels of microfluidic cell culture devices, it is necessary to adopt an approach which bypasses the need for baseline subtraction typically seen with single (12.5 Hz) frequency-based impedance measurements. Using a compensation method capable of correcting high frequency parasitics, we demonstrated the suitability of frequency sweep impedance measurements and analog fitting for on-a-Chip TEER applications. Finally, the absence of any membranes, filters or other barriers supporting the cells avoids the potential bias caused by pore size and geometry. These properties combined with excellent imaging and automation compatibility make this platform the ideal candidate for transport and barrier integrity studies.

Helper T cells, specifically Th1 cells, are often found in atherosclerotic lesions and secrete INF-γ and TNFα, which both promote inflammation in endothelial cells (45–47). INF-γ showed a slow dose dependent decrease in endothelial barrier over the 48 hours but did not have a significant change in VE-Cadherin. Minagar et al. and Oshima et al. found that INF-γ caused a significant decrease in endothelial barrier and a small decrease in VE-Cadherin expression (48, 49). Interestingly, we did not observe a statistically significant increase in ICAM-1 expression after INF-γ exposure. In literature, it has been shown that INF-γ can cause an increase in ICAM-1 expression in some endothelial cells like HUVECs but not in other endothelial cell lines (50). However, this difference was observed using a monolayer of endothelial cells whereas the OrganoPlate is an organ-on-a-chip platform with perfusion which could lead to the difference in ICAM-1 expression.

TNFα had a significant dose dependent decrease in barrier but seemed to recover by the end of 48 hours. Using this platform, we were able to see the dynamic response TNFα had on the barrier that would have been missed with single timepoint measurements. It is interesting that the barrier appeared to recover at later time points because there was a significant change in cell morphology and appearance of VE-Cadherin expression. Similarly, Colas-Algora et al. show that TNFα causes a decrease TEER and VE-Cadherin reorganizing in HUVECs (51). TNFα did cause a significant increase in ICAM expression, which is consistent with literature (52, 53). Exposure to both cytokines together did appear to have an additive effect which greater changes in VE-Cadherin and ICAM expression. The change in barrier function showed both the sharp decrease in TEER associated with TNFα, and the slow constant decrease associated with INF-γ. These additive effects are in line with the exquisite control of inflammatory response achieved by complex mixtures of cytokines in physiological processes. Interestingly, the stimulated T cells had similar changes in barrier, ICAM expression and changes endothelial cell morphologies to the TNFα and INF-γ condition. Therefore, this data shows that vascular inflammation can be recapitulated using either inflammatory cytokines or activated immune cells.

Previously, we have reported the transendothelial migration of T cells from the endothelial tube into the collagen gel in the OrganoPlate (26). As with the results previously shown, both cytokines and a chemoattractant must be present for the migration of unstimulated immune cells, which is similar to *in vivo* settings (54). The unstimulated immune cells alone had no effect on the barrier, ICAM expression or migration but the addition of both 10 ng/ml of INF-γ and TNFα with CLCX12, there was significant migration into the ECM. Interestingly, the conditions with and without CLCX12 did not have any significant changes in barrier. Indicating that the migrating immune cells most likely have minimal effect on endothelial barrier, and/or that our TEER measurements were not sensitive enough to detect the slight changes in barrier. The unstimulated PBMCs did not have an effect on endothelial cell roundness but the stimulated PBMCs did have a significant decrease in roundness of the endothelial cells. The addition of cytokines to all conditions had the greatest effect on cell roundness, which indicates that cytokines could be a relevant trigger to model vascular inflammation in a simplistic model. This platform allows for the study of the effects of cytokines and chemoattractants on both immune cells and endothelial cells individually and in combination.

Here we used the addition of inflammatory cytokines and immune cells to model vascular inflammation looking at changes in barrier permeability over time, different inflammatory markers and adherent junctions and quantification of immune cell adhesion and migration. In addition to modeling vascular inflammation, the TEER timelapse could potentially be used to improve the blood brain barrier models (30, 55), immune-oncology (26), and many other diseases. Overall, the OrganoPlate 3-lane 64 is a scalable, automation compatible tool that allows for multiplexing of assays to be able to do complex disease modeling for drug development.

## Data availability statement

The raw data supporting the conclusions of this article will be made available by the authors, without undue reservation.

## Author contributions

HE and AN contributed equally in preparation of the manuscript. AN and ST invented the OrganoTEER concept. AN, FS, MB, and ST contributed to the design and engineering of the instrument and software. HE, JH, AN, and LB planned, performed, and analyzed experiments. ST and LB supervised the work and edited the manuscript. All authors contributed to the article and approved the submitted version.

## References

[1] Pahwa R Goyal A Jialal I. Chronic inflammation. In: Pathobiol hum dis a dyn encycl dis mech (2022). [Book StatPearls: StatPearls Publishing, Treasure Island (FL)] Available at: https://www.ncbi.nlm.nih.gov/books/NBK493173/.

[2] Zanoli L Briet M Empana JP Guimarães Cunha P Mäki-Petäjä M Protogerou AD. Vascular consequences of inflammation: A position statement from the ESH working group on vascular structure and function and the ARTERY society. J Hypertens (2020) 38(9):1682–98. doi: 10.1097/HJH.0000000000002508 PMC7610698 32649623

[3] Castellon X Bogdanova V. Chronic inflammatory diseases and endothelial dysfunction. Aging Dis (2016) 7 (1):1–89. doi: 10.14336/AD.2015.0803 26815098 PMC4723236

[4] Cardiovascular diseases . Available at: https://www.who.int/health-topics/cardiovascular-diseases#tab=tab_1.

[5] Xu S Ilyas I Little PJ Li H Kamato D Zheng X. Endothelial dysfunction in atherosclerotic cardiovascular diseases and beyond: From mechanism to pharmacotherapies. Pharmacol Rev (2021) 73(3):924–67. doi: 10.1124/pharmrev.120.000096 34088867

[6] Rafieian-Kopaei M Setorki M Doudi M Baradaran A Nasri H. Atherosclerosis: Process, indicators, risk factors and new hopes. Int J Prev Med (2014) 5(8):927–946. PMC4258672 25489440

[7] McDonald DM. Angiogenesis and remodeling of airway vasculature in chronic inflammation. Am J Respir Crit Care Med (2001) 164(10 Pt 2):S39-S45. doi: 10.1164/ajrccm.164.supplement_2.2106065 11734465

[8] Claesson-Welsh L Dejana E McDonald DM. Permeability of the endothelial barrier: Identifying and reconciling controversies. Trends Mol Med (2021) 27(4):314–31. doi: 10.1016/j.molmed.2020.11.006 PMC8005435 33309601

[9] Junaid A Schoeman J Yang W Stam W Mashaghi A Van Zonneveld AJ. Metabolic response of blood vessels to TNFα. eLife (2020) 9:1–17. doi: 10.7554/eLife.54754 PMC7476757 32749215

[10] Rajan S Ye J Bai S Huang F Guo YL. NF-κB, but not p38 MAP kinase, is required for TNF-α–induced expression of cell adhesion molecules in endothelial cells. J Cell Biochem (2008) 105(2):477. doi: 10.1002/jcb.21845 18613029 PMC4422387

[11] Gavard J. Endothelial permeability and VE-cadherin. Cell Adh Migr (2013) 7(6):465–71. doi: 10.4161/cam.27330 PMC3916348 24430214

[12] Harris ES Nelson J. VE-cadherin: at the front, center, and sides of endothelial cell organization and function (2022). Available at: www.sciencedirect.com. 10.1016/j.ceb.2010.07.006 PMC2948582 20708398

[13] Sluiter TJ van Buul JD Huveneers S Quax PHA de Vries MR. Endothelial barrier function and leukocyte transmigration in atherosclerosis. Biomedicines (2021) 9(4):328. doi: 10.3390/biomedicines9040328 33804952 PMC8063931

[14] Allingham MJ Van Buul JD Burridge K. Transendothelial migration phosphorylation is required for leukocyte vascular endothelial cadherin tyrosine ICAM-1-Mediated, src-and Pyk2-dependent. J Immunol Ref (2007) 179:4053–64. doi: 10.4049/jimmunol.179.6.4053 17785844

[15] Turowski P Martinelli R Crawford R Wateridge D Papageorgiou AP Lampugnani MG. Phosphorylation of vascular endothelial cadherin controls lymphocyte emigration. J Cell Sci (2008) 121(1):29–37. doi: 10.1242/jcs.022681 18096689 PMC3810954

[16] Broermann A Winderlich M Block H Frye M Rossaint J Zarbock A. Dissociation of VE-PTP from ve-cadherin is required for leukocyte extravasation and for VEGF-induced vascular permeability in vivo. J Exp Med (2011) 208(12):2393–401. doi: 10.1084/jem.20110525 PMC3256962 22025303

[17] Heemskerk N Schimmel L Oort C Van Rijssel J Yin T Ma B. F-actin-rich contractile endothelial pores prevent vascular leakage during leukocyte diapedesis through local RhoA signalling. Nat Commun (2016) 7:10493. doi: 10.1038/ncomms10493 26814335 PMC4737874

[18] Sun W Chen YQ Luo GA Zhang M Zhang HY Wang YR. Organs-on-chips and its applications. Chin J Anal Chem (2016) 44(4):533–41. doi: 10.1016/S1872-2040(16)60920-9

[19] Huh D Hamilton GA Ingber DE. From 3D cell culture to organs-on-chips. Trends Cell Biol (2011) 21(12):745–54. doi: 10.1016/j.tcb.2011.09.005 PMC4386065 22033488

[20] Doherty EL Aw WY Hickey AJ Polacheck WJ. Microfluidic and organ-on-a-Chip approaches to investigate cellular and microenvironmental contributions to cardiovascular function and pathology. Front Bioeng Biotechnol (2021) 9(February):1–14. doi: 10.3389/fbioe.2021.624435 PMC7890362 33614613

[21] Partyka PP Godsey GA Galie JR Kosciuk MC Acharya NK Nagele RG. Mechanical stress regulates transport in a compliant 3D model of the blood-brain barrier. Biomaterials (2017) 115:30–9. doi: 10.1016/j.biomaterials.2016.11.012 27886553

[22] Polacheck WJ Kutys ML Tefft JB Chen CS. Microfabricated blood vessels for modeling the vascular transport barrier Vol. Vol. 14. . Springer US: Nature Protocols (2019) p. 1425–54. doi: 10.1038/s41596-019-0144-8 PMC7046311 30953042

[23] Kim S Kim W Lim S Jeon JS. Vasculature-on-a-chip for in vitro disease models. Bioengineering (2017) 4(4):8. doi: 10.3390/bioengineering4010008 28952486 PMC5590435

[24] Ingber DE. Human organs-on-chips for disease modelling, drug development and personalized medicine. Nat Rev Genet (2022) 23(August):467–91. doi: 10.1038/s41576-022-00466-9 PMC8951665 35338360

[25] Van Duinen V Van Den Heuvel A Trietsch SJ Lanz HL Van Gils JM Van Zonneveld AJ. 96 perfusable blood vessels to study vascular permeability in vitro. Sci Rep (2017) 7(1):1–11. doi: 10.1038/s41598-017-14716-y 29273771 PMC5741747

[26] de Haan L Suijker J van Roey R Berges N Petrova E Queiroz K. A microfluidic 3D endothelium-on-a-chip model to study transendothelial migration of T cells in health and disease. Int J Mol Sci (2021) 22(15):8234. Available at: 10.3390/ijms22158234 34361000 PMC8347346

[27] Poussin C Kramer B Lanz HL van den Heuvel A Laurent A Olivier T. 3D human microvessel-on-a-chip model for studying monocyte-to-endothelium adhesion under flow – application in systems toxicology. ALTEX - altern to anim exp (2020). Available at: https://www.altex.org/index.php/altex/article/view/1212. 10.14573/altex.1811301 31445503

[28] Nicolas A Schavemaker F Kosim K Kurek D Haarmans M Bulst M. High throughput transepithelial electrical resistance (TEER) measurements on perfused membrane-free epithelia. Lab Chip (2021) 21(9):1676–85. doi: 10.1039/D0LC00770F 33861225

[29] Vulto P Podszun S Meyer P Hermann C Manz A Urban GA. Phaseguides: A paradigm shift in microfluidic priming and emptying. Lab Chip. (2011) 11(9):1596–602. doi: 10.1039/c0lc00643b 21394334

[30] Wevers NR Kasi DG Gray T Wilschut KJ Smith B Vught R. A perfused human blood-brain barrier on-a-chip for high-throughput assessment of barrier function and antibody transport. Fluids Bar. CNS (2018) 15(1):1–12. doi: 10.1186/s12987-018-0108-3 PMC6117964 30165870

[31] Riddle RB Jennbacken K Hansson KM Harper MT. Endothelial inflammation and neutrophil transmigration are modulated by extracellular matrix composition in an inflammation-on-a-chip model. Sci Rep (2022) 12(1):1–14. doi: 10.1038/s41598-022-10849-x 35477984 PMC9046410

[32] Kramer B Corallo C van den Heuvel A Crawford J Olivier T Elstak E. High-throughput 3D microvessel-on-a-chip model to study defective angiogenesis in systemic sclerosis. Sci Rep (2022) 12(1):1–12. doi: 10.1038/s41598-022-21468-x 36209279 PMC9547891

[33] Eigenmann DE Xue G Kim KS Moses AV Hamburger M Oufir M. Comparative study of four immortalized human brain capillary endothelial cell lines, hCMEC/D3, hBMEC, TY10, and BB19, and optimization of culture conditions, for an *in vitro* blood-brain barrier model for drug permeability studies. Fluids Bar. CNS (2013) 10(1):1–17. doi: 10.1186/2045-8118-10-33 PMC4176484 24262108

[34] Von Wedel-Parlow M Wölte P Galla HJ. Regulation of major efflux transporters under inflammatory conditions at the blood-brain barrier in vitro. J Neurochem (2009) 111(1):111–8. doi: 10.1111/j.1471-4159.2009.06305.x 19656257

[35] Tiruppathi C Malik AB Del Vecchio PJ Keese CR Glaever I. Electrical method for detection of endothelial cell shape change in real time: assessment of endothelial barrier function. Proc Natl Acad Sci (1992) 89(17):7919–23. doi: 10.1073/pnas.89.17.7919 PMC49826 1518814

[36] Benson K Cramer S Galla HJ. Impedance-based cell monitoring: Barrier properties and beyond. Fluids Bar. CNS (2013) 10(1):1–11. doi: 10.1186/2045-8118-10-5 PMC3560213 23305242

[37] Srinivasan B Kolli AR Esch MB Abaci HE Shuler ML Hickman JJ. TEER measurement techniques for *In vitro* barrier model systems. SLAS Technol (2015) 20(2):107–26. doi: 10.1177/2211068214561025 PMC4652793 25586998

[38] Naumovska E Aalderink G Valencia CW Kosim K Nicolas A Brown S. Direct on-chip differentiation of intestinal tubules from induced pluripotent stem cells. Int J Mol Sci (2020) 21:4964. doi: 10.3390/ijms21144964 32674311 PMC7404294

[39] Beaurivage C Naumovska E Chang YX Elstak ED Nicolas A Wouters H. Development of a gut-on-a-Chip model for high throughput disease modeling and drug discovery. Int J Mol Sci (2019) 20:5661. doi: 10.3390/ijms20225661 31726729 PMC6888156

[40] Bircsak KM DeBiasio R Miedel M Alsebahi A Reddinger R Saleh A. A 3D microfluidic liver model for high throughput compound toxicity screening in the OrganoPlate^®^ . Toxicology (2021) 450:152667. doi: 10.1016/j.tox.2020.152667 33359578

[41] van Duinen V Zhu D Ramakers C van Zonneveld AJ Vulto P Hankemeier T. Perfused 3D angiogenic sprouting in a high-throughput in vitro platform.. Angiogenesis (2019) 22(1):157–65. doi: 10.1007/s10456-018-9647-0 PMC6510881 30171498

[42] Wevers NR Van Vught R Wilschut KJ Nicolas A Chiang C Lanz HL. High-throughput compound evaluation on 3D networks of neurons and glia in a microfluidic platform. Sci Rep (2016) 6(November):1–10. doi: 10.1038/srep38856 27934939 PMC5146966

[43] Stringer C Pachitariu M. Cellpose 2.0: how to train your own model. Nat Methods 19:1634–1641 (2022) doi: 10.1101/2022.04.01.486764 36344832 PMC9718665

[44] Schindelin J Arganda-Carreras I Frise E Kaynig V Longair M Pietzsch T. Fiji - an open source platform for biological image analysis. Nat Methods (2012) 9(7):676–82. doi: 10.1038/nmeth.2019 PMC3855844 22743772

[45] Moriya J. Critical roles of inflammation in atherosclerosis. J Cardiol (2019) 73(1):22–7. doi: 10.1016/j.jjcc.2018.05.010 29907363

[46] Nakiboneka R Mugaba S Auma BO Kintu C Lindan C Nanteza MB. Interferon gamma (IFN-γ) negative CD4+ and CD8+ T-cells can produce immune mediators in response to viral antigens. Vaccine (2019) 37(1):113. doi: 10.1016/j.vaccine.2018.11.024 30459072 PMC6290111

[47] Rodrigues LS Barreto AS Bomfim LGS Gomes MC Ferreira NLC da Cruz GS. Multifunctional, TNF-α and IFN-γ-Secreting CD4 and CD8 T cells and CD8High T cells are associated with the cure of human visceral leishmaniasis. Front Immunol (2021) 12(October):1–20. doi: 10.3389/fimmu.2021.773983 PMC8581227 34777391

[48] Minagar A Long A Ma T Jackson TH Kelley RE Ostanin DV. Interferon (IFN)-ß1a and IFN-ß1b block IFN-?-Induced disintegration of endothelial junction integrity and barrier. Endothelium (2009) 10(6):299–307. doi: 10.1080/10623320390272299 14741845

[49] Oshima T Laroux FS Coe LL Morise Z Kawachi S Bauer P. Interferon-γ and interleukin-10 reciprocally regulate endothelial junction integrity and barrier function. Microvasc Res (2001) 61(1):130–43. doi: 10.1006/mvre.2000.2288 11162203

[50] Thornhill MH Li J Haskard DO. Leucocyte endothelial cell adhesion: a study comparing human umbilical vein endothelial cells and the endothelial cell line EA-hy-926. Scand J Immunol (1993) 38(3):279–86. doi: 10.1111/j.1365-3083.1993.tb01726.x 8356403

[51] Colás-Algora N García-Weber D Cacho-Navas C Barroso S Caballero A Ribas C. Compensatory increase of VE-cadherin expression through ETS1 regulates endothelial barrier function in response to TNFα. Cellular and Molecular Life Sciences (2020) 77:2125–40. doi: 10.1007/s00018-019-03260-9 PMC11105044 31396656

[52] Yang L Froio RM Sciuto TE Dvorak AM Alon R Luscinskas FW. ICAM-1 regulates neutrophil adhesion and transcellular migration of TNF-activated vascular endothelium under flow (2005). Available at: http://ashpublications.org/blood/article-pdf/106/2/584/1711514/zh801405000584.pdf. 10.1182/blood-2004-12-4942 PMC1635241 15811956

[53] Jiang Y Jiang LLI Maimaitirexiati XMZY Zhang Y Wu L. Irbesartan attenuates TNF-α-induced ICAM-1, VCAM-1, and e-selectin expression through suppression of NF-κ b pathway in HUVECs. Eur Rev Med Pharmacol Sci (2015) 19(17):3295–302. 26400537

[54] Reiss Y Engelhardt B. T Cell interaction with ICAM-1-deficient endothelium *in vitro*: transendothelial migration of different T cell populations is mediated by endothelial ICAM-1 and ICAM-2. Int Immunol (1999) 11(9):1527–39. doi: 10.1093/intimm/11.9.1527 10464174

[55] Wevers NR Nair AL Fowke TM Pontier M Kasi DG Spijkers XM. Modeling ischemic stroke in a triculture neurovascular unit on-a-chip. Fluids Bar. CNS (2021) 18(1):1–18. doi: 10.1186/s12987-021-00294-9 PMC8670153 34906183

