# Supplementary material for: Vascular inflammation on a chip: A scalable platform for trans-endothelial electrical resistance and immune cell migration
Source: Front Immunol. 2023 Jan 24;14:1118624. doi: 10.3389/fimmu.2023.1118624 (PMC9903066; doi:10.3389/fimmu.2023.1118624)
Supplement: Supplementary file 1 [file DataSheet_1.pdf]

Supplemental Materials

Vascular inflammation on a chip: a scalable platform for Trans-Endothelial Electrical Resistance and immune cell migration

H. Ehlers, A. Nicolas, F. Schavemaker, J. Heijmans, M. Bulst, S.J. Trietsch, L.J. van den Broek

*** Correspondence:** Lenie van den Broek, l.vandenbroek@mimetas.com

OrganoTEER 3 lane 64 electrode board compensation

To compensate for the parallel capacitive parasitic component affecting the higher frequency portion (10 kHz-150 kHz) of the measured impedance spectra, we built a compensation method using three resistive load boards and a first order polynomial fitting model to correct each measurement point frequency dependent response.

Each of the compensation board is built by soldering a surface mounted resistor of defined value (30 kΩ, 33 kΩ, 36 kΩ) for each of the 64 four-point measurement circuits. The resistor shorts together the reference – counter ports and working - working sense ports respectively. By measuring these three compensations boards, we capture the parasitic elements associated with the measurement unit and PCB traces.

For each of the $n = 121$ measured impedance frequency point, we fit the ratio of first order polynomial function

$$H(f) = \frac{a_f + b_f * Z(f)}{1 + c_f * Z(f)}$$

Where $Z(f)$ if the complex impedance measured at frequency f, $H(f)$ is the associated compensation function and (a_f, b_f, c_f) are the $nx3$ parameters to estimate from the compensation measurements.

Since the model has three unknown parameters per measured frequency points, three compensation spectra allow us to estimate the full compensation model. We chose resistor values at and below the typical value of a cell free control (36 kΩ).

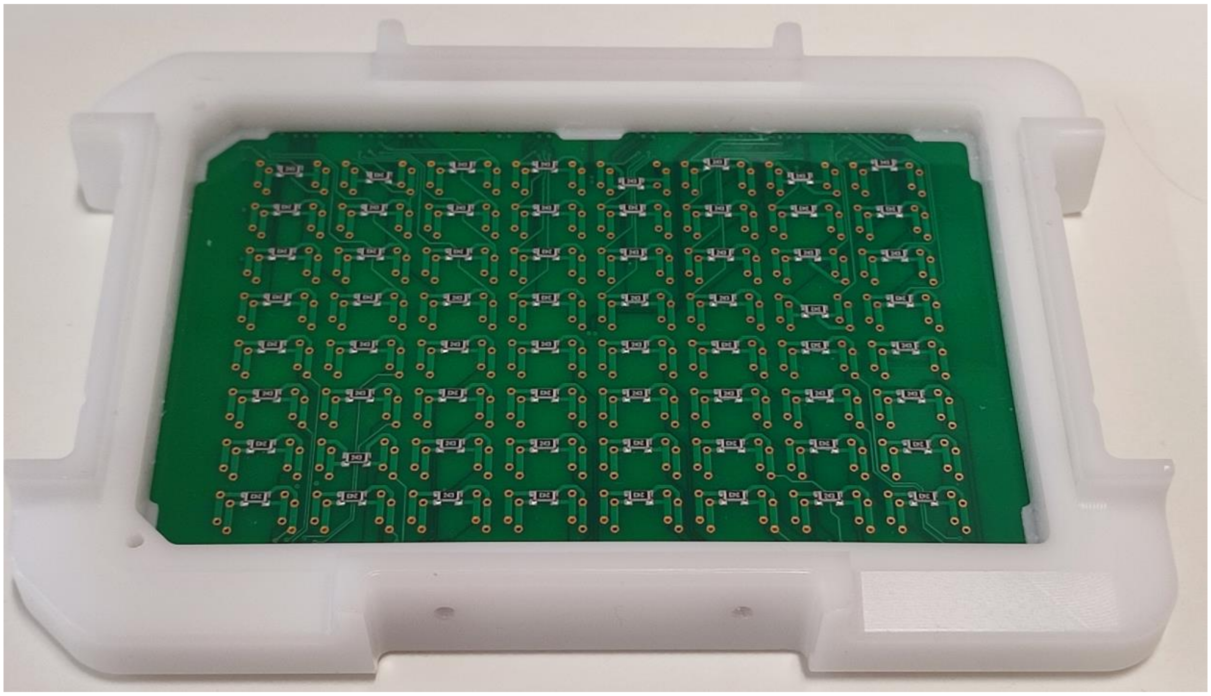

Supplementary Figure 1. Compensation board with surface mounted resistors. The resistors loads are measured on a PCB designed to reproduce the 3 lane 64 electrode board geometry.

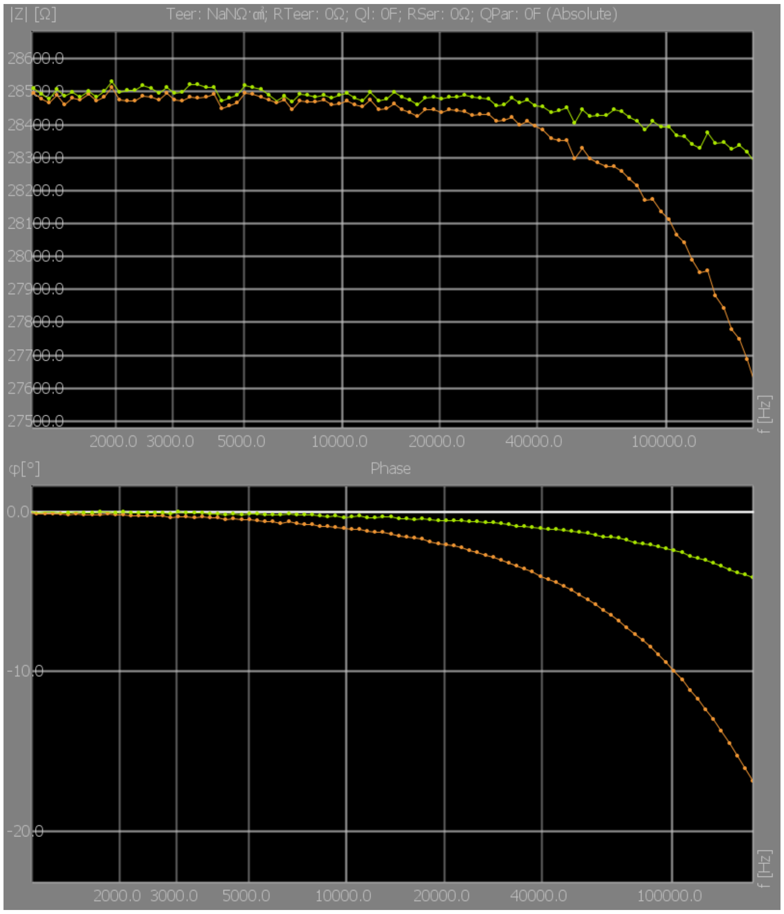

Supplementary Figure.2. Bode plot of a measured spectrum on a control chip without cells. In red, the uncorrected spectrum exhibits parasitic capacitive effects in high frequency. After compensation, the spectrum (green) shows lower parasitic capacitance.

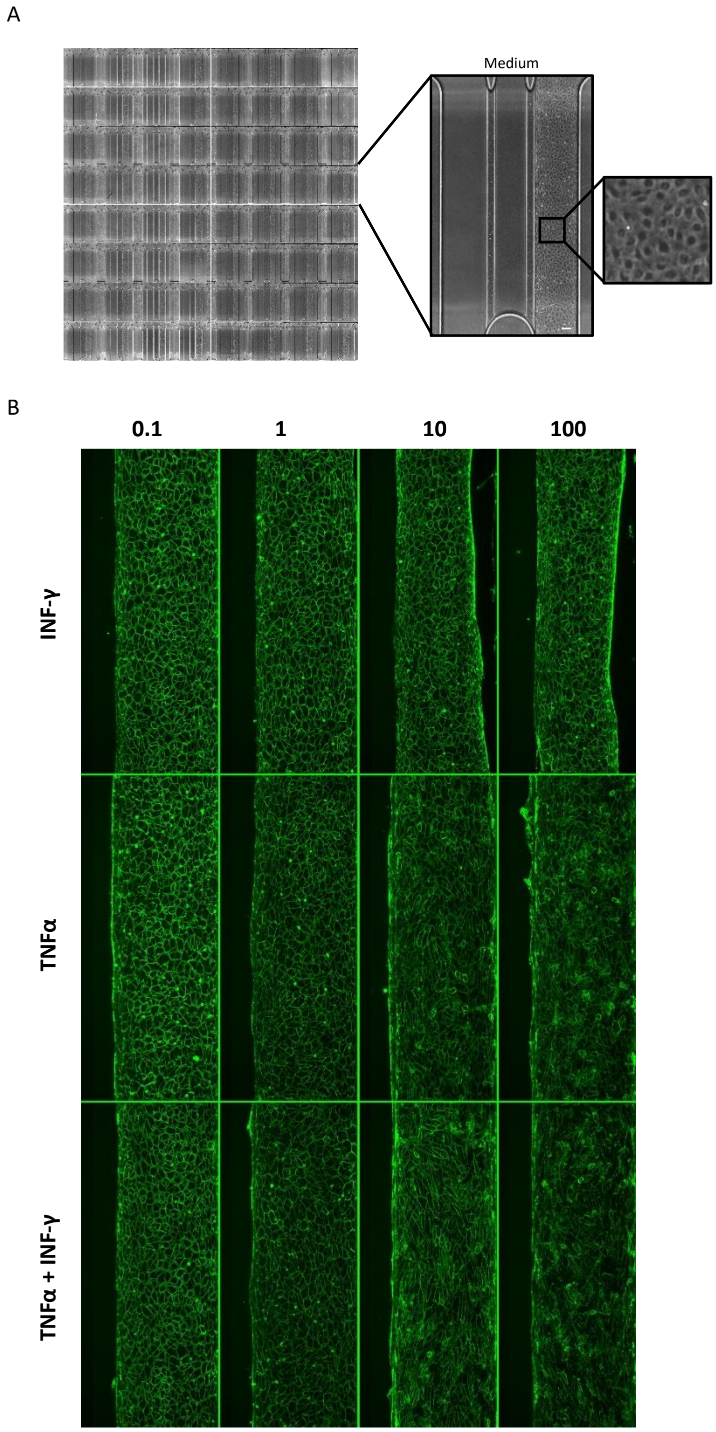

Supplementary Figure 3. Additional images of cytokine response of HUVEC endothelial tubes in the OrganoPlate 3-lane 64. **(A)** Phase contrast montage of 3-lane 64 OrganoPlate highlighting one chip and zoomed in of the endothelial tube. **(B)** Max projection of bottom 10 z-steps (3µm step size) of VE-cadherin expression to highlight differences in VE-Cadherin across the whole vessel. Concentration of cytokines in ng/ml.

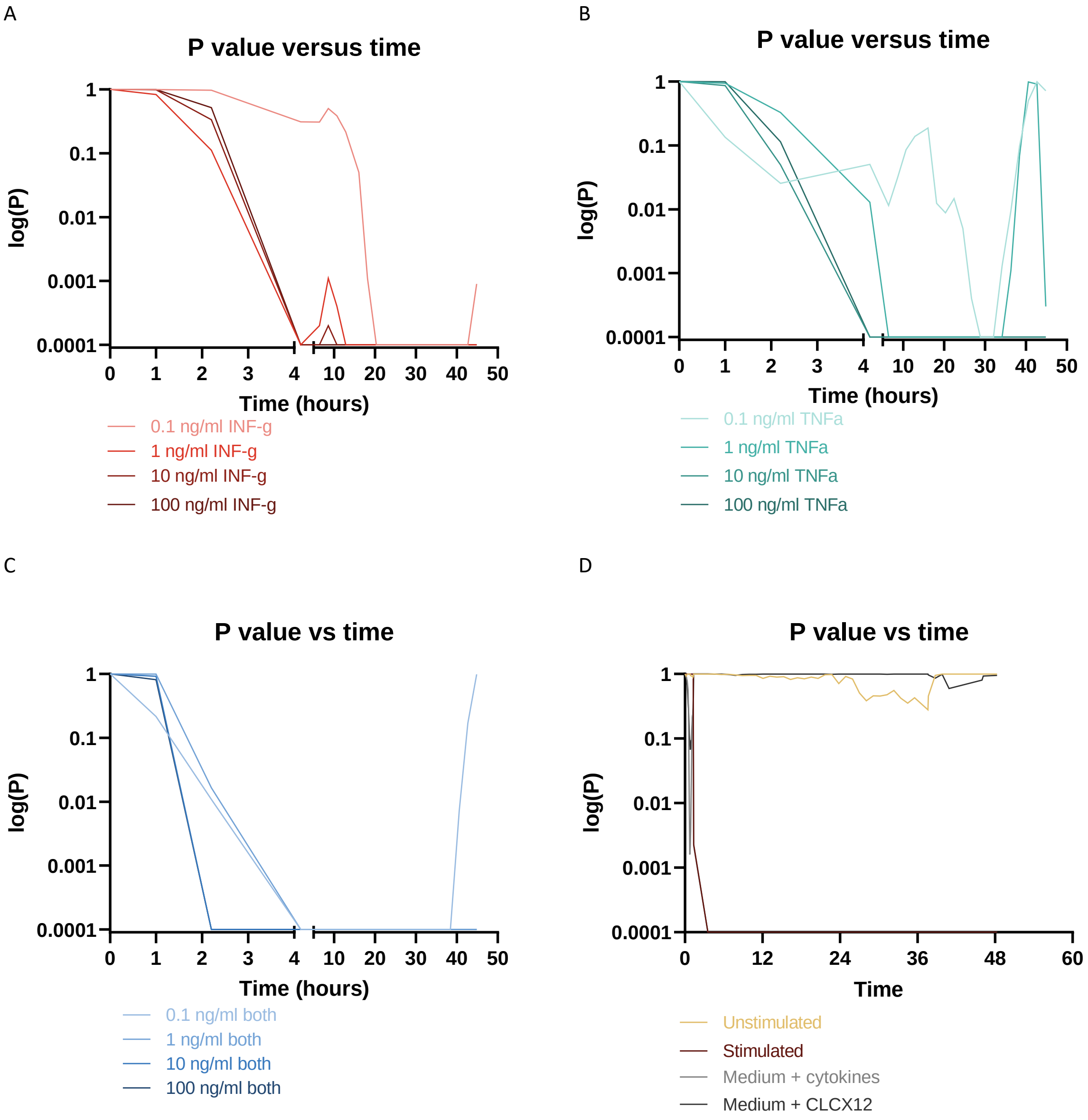

Supplementary Figure 4. TEER statistics showing log(P) versus time for each cytokine condition over time. **(A)** Change in INF-γ P value over time. **(B)** Change in TNFα P value over time. **(C)** Change in TNFα + INF-γ P value over time. **(D)** Change in P value over time due to addition of unstimulated/stimulated PBMCs compared to medium with cytokine or CLCX12.

a.

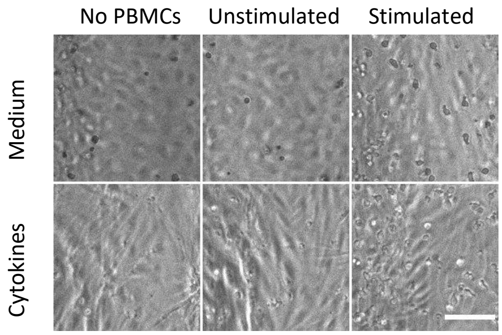
No PBMCs
Unstimulated
Stimulated
Medium
Cytokines

b.

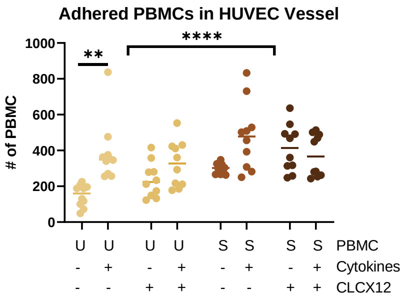
Adhered PBMCs in HUVEC Vessel
# of PBMC
1000
800
600
400
200
0
**
****
U U U U S S S S PBMC
- + - + - + - + Cytokines
- - + + - - + + CLCX12

c.

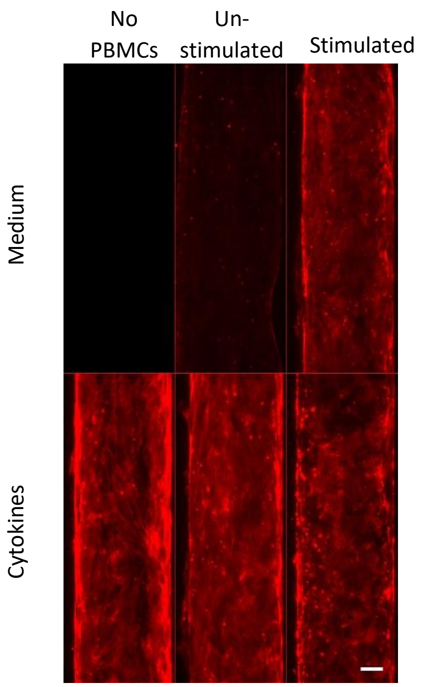
No PBMCs
Un-stimulated
Stimulated
Medium
Cytokines

d.

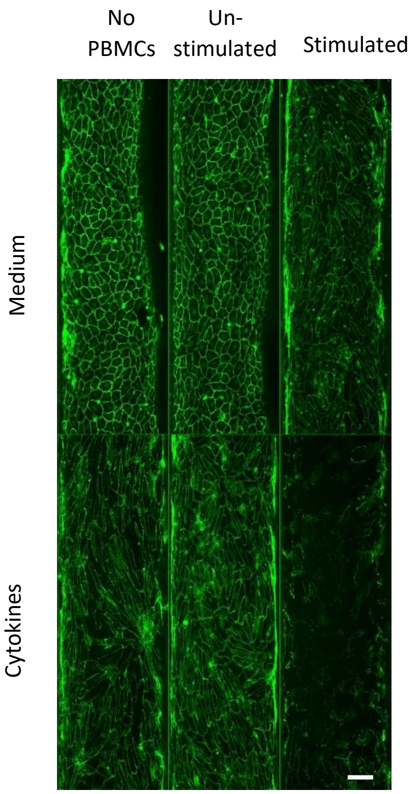
No PBMCs
Un-stimulated
Stimulated
Medium
Cytokines

e.

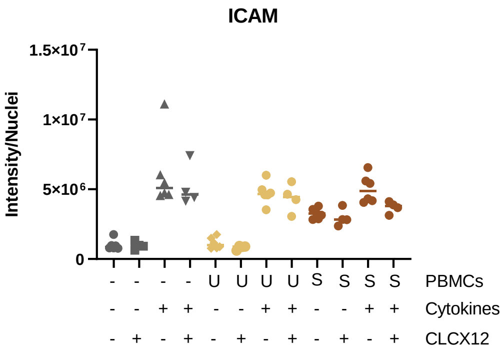
ICAM
Intensity/Nuclei
1.5×10^7
1×10^7
5×10^6
0
- - - - U U U U S S S S PBMCs
- - + + - - + + - - + + Cytokines
- + - + - + - + - + - + CLCX12

f.

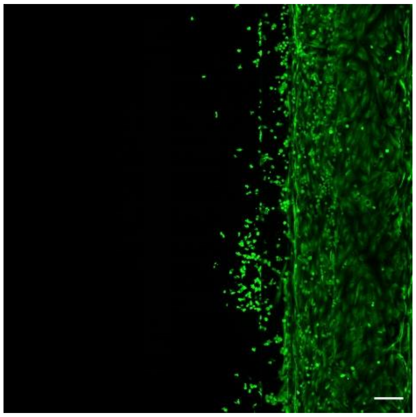

Supplementary Figure 5. Additional images and quantification of perfusion and extravasation of peripheral blood mononuclear cells (PBMCs) through a HUVEC tubule into an ECM gel in an OrganoPlate 64. **(a)** Phase contrast montage of endothelial vessel +/- cytokines and +/- Unstimulated or Stimulated PBMCs. **(b)** Quantification of adhered PBMCs in vessel. Significant difference between unstimulated PBMC and stimulated PBMCs (**** $p < 0.0001$) were analyzed using 3way ANOVA test. Significant different in unstimulated PBMCs with cytokines versus no cytokine addition (** $p=0.0046$) were analyzed using a Brown-Forsythe and Welch ANOVA tests with Dunnett's T3 multiple comparison. **(c)** Montage of SUM projection of ICAM-1. **(d)** Max projection of bottom 10 z-steps (3µm step size) of VE-cadherin expression. **(e)** No effect of CXCL12 addition to the cultures in ICAM-1 expression. ICAM-1 is shown as total intensity / nuclei. expression due to addition of CLCX12. Statistics need to be finished but will use a 3way ANOVA to show ns for addition of CLCX12. **(f)** Max projection of calcein staining assay showing PBMCs are still viable in the gel following TEER measurements. Scale bars are 100µm.
